# Gut microbiome associated with melanin deposition by supporting energy metabolism in Sichuan mountainous black-bone chickens

**DOI:** 10.3389/fmicb.2025.1682376

**Published:** 2025-12-15

**Authors:** Juan Liao, Ling Duan, Gang Wang, Shigang Yu, Xuemei Shen, Min Jiang, Ke Shen, Rajeev K. Singla, Bairong Shen, Yi Zhou

**Affiliations:** 1 Engineering Research Center of Sichuan Province Higher School of Local Chicken Breeds Industrialization in Southern Sichuan, College of Life Science, Leshan Normal University, Leshan, China; 2 Sichuan Academy of Chinese Medicine Sciences, Chengdu, China; 3 Central Station of Animal Feed Affairs of Sichuan Province, Sichuan Provincial Department of Agriculture and Rural Affairs, Chengdu, China; 4 Department of Critical Care Medicine, Center for High Altitude Medicine, Institutes for Systems Genetics, Frontiers Science Center for Disease-related Molecular Network, West China Hospital, Sichuan University, Chengdu, China; 5 School of Pharmaceutical Sciences, Lovely Professional University, Phagwara, Punjab, India

**Keywords:** Sichuan mountainous black-bone chicken, gut microbiome, untargeted-metabolome, pigmentation, melanin

## Abstract

**Introduction:**

Variation in melanin deposition profoundly influences the economic value of Sichuan mountainous black-boned chickens; however, the contribution of the gut microbiome in modulating this process remains poorly understood. This study aimed to characterize the gut microbiome in Sichuan mountainous black-boned chickens with distinct skin color brightness (quantified by colorimeter) and to explore its association with melanin deposition.

**Methods:**

Cecal microbiota composition was profiled using 16S rRNA sequencing in dark-skinned (BlackD) and light-skinned (BlackL) groups. Serum metabolic profiles were determined via untargeted metabolomics. Differential abundance of bacterial genera was assessed, followed by pathway enrichment analysis (KEGG and MetaCyc). Associations between microbiome, metabolites, and melanin-related pathways were evaluated.

**Results:**

The BlackD group exhibited higher alpha diversity and significant alterations in 10 bacterial genera (primarily linked to short-chain fatty acid metabolism and melanin-related metabolites) compared to the BlackL group. Pathway enrichment showed upregulation of energy metabolism-related KEGG pathways (AMPK signaling, insulin signaling, thyroid hormone signaling) and MetaCyc pathways in BlackD. Untargeted serum metabolomics revealed elevated melanin-related metabolites in BlackD, including L-Tyrosine, L-DOPA, and Dopaquinone. Gut microbiome and serum metabolite profiles in BlackD were strongly correlated with enhanced energy metabolism.

**Discussion:**

The gut microbiome may influence melanin deposition by modulating host metabolic activity, with microbiome-derived metabolites supporting the high energy demands of melanocyte activity. These findings uncover a potential mechanism linking microbial composition to phenotypic variation in melanin deposition.

## Introduction

The Sichuan mountainous black-bone chicken, an indigenous poultry breed originating from Muchuan County, Sichuan Province, has been cultivated for centuries (Liao et al., 2024). Renowned for its distinctive black pigmentation, observed in the skin, comb, beak, muscles, viscera, and bones, this breed holds dual significance in both culinary traditions and traditional Chinese medicine due to its purported medicinal properties (Jian et al., 2021; Tian et al., 2007). The feature of black bone chickens lies in their elevated melanin content, which confers biological benefits, including free radical scavenging, antioxidant activity, anti-aging effects, anti-mutagenic potential, and immune enhancement (Jian et al., 2021; Sehrawat et al., 2021). Additionally, these chickens are rich in protein, calcium, iron, zinc, and essential trace elements, contributing to their exceptional nutritional profile and widespread consumer appeal (Jian et al., 2021; Liao et al., 2022; Nganvongpanit et al., 2020). Notably, darker skin pigmentation is strongly associated with perceived quality and therapeutic value (Wang et al., 2018). However, under standardized breeding conditions, phenotypic variability in skin coloration persists within populations, with lighter-skinned individuals exhibiting lower market value.

Variation in melanin deposition in black bone chickens arises from a complex interplay of genetic, nutritional, and environmental factors (Li et al., 2024; Wang et al., 2018). For instance, in the Oujiang color common carp, mutations in the *SCARB1* gene result in a white phenotype compared to the wild type (Nathan Mandal et al., 2024), while *TYRP1* mutations lead to a coffee-colored variant (Kanika et al., 2024). Intriguingly, these mutations are accompanied by significant differences in gut microbiota composition. In *SCARB1* mutants, microbial taxa associated with lipase production, such as *Bacillus*, *Staphylococcus*, *Pseudomonas*, and *Serratia*, exhibit significantly reduced abundance (Nathan Mandal et al., 2024). Conversely, genera such as *Acinetobacter* and *Achromobacter* (Proteobacteria), as well as *Flavobacterium* and *Terrimonas* (Bacteroidetes), are only detected in *TYRP1* mutants (Kanika et al., 2024). Similarly, in Jiangshan black bone chickens, the relative abundance of *Escherichia-Shigella* is notably higher in the black group (25.88%) compared to the green (0.18%) and blue (0.86%) groups (Wang et al., 2024). Indeed, melanin deposition is a tightly regulated biochemical process that extends beyond enzymatic modifications (e.g., tyrosinase-mediated oxidation) (Snyman et al., 2024). It requires the orchestrated involvement of molecular chaperones, transporters, and structural proteins, alongside metabolic energy, to facilitate the synthesis, transport, and localization of melanin granules (Daniele et al., 2014; Snyman et al., 2024). The diverse metabolic molecules and energy substrates essential for this process can be supplied by the complex metabolites produced by the gut microbiome (Heiss and Olofsson, 2018; Turner et al., 2023). This evidence suggests that the gut microbiome plays an important role in influencing host pigmentation.

Despite these advances, the relationship between gut microbiome and melanin pigmentation in Sichuan mountainous black-bone chickens remains largely unexplored. This study employs 16S rRNA sequencing and untargeted metabolomics to compare gut microbial diversity and serum metabolite profiles between dark-skinned and light-skinned Sichuan mountainous black-bone chickens. Our objective is to investigate the potential link between gut microbiome and skin pigmentation, thereby providing new insights into the mechanisms underlying pigmentation variation in this economically valuable indigenous breed.

## Materials and methods

### Chickens and sample collection

Sichuan mountainous black-bone chicken was provided by Heifenghuang Black-bone Chicken Industry Co., Ltd. (Muchuan County, Sichuan Province, China). All birds were raised under identical feeding regimes and management conditions to ensure experimental consistency. The study was supervised by the Animal Ethics Committee of Leshan Normal University (No. 2022091132).

A total of 148 healthy female Sichuan mountain black-bone chickens, aged 150 days and with comparable body weights, were randomly selected. Skin color was measured at four anatomical regions: dorsal surface (back), mid-joint wing (wing), comb, and thigh, using a CIELab-calibrated portable colorimeter (NR10QC; 3nh Technologies). Statistical analysis (Table 1) revealed that the dorsal surface exhibited the most pronounced difference in brightness values. Based on this, the chickens were classified into two groups: a dark-skinned group (BlackD) and a light-skinned group (BlackL), with 12 chickens in each group. Aseptic procedures were applied during sample collection. Cecal contents and serum samples were collected from each bird in the BlackD and BlackL groups. Individuals were sequentially numbered as BlackD1-BlackD12 and BlackL1-BlackL12 (serum samples were collected for BlackD1-D8 and BlackL1-L8). All samples were immediately snap-frozen in liquid nitrogen and subsequently stored at −80°C until further analysis.

**TABLE 1:** Results of skin color determination in different regions of Sichuan mountainous black-bone chicken.

Measurement	Items	Mean-BlcakD	SE-BlackD	Mean-BlcakL	SE-BlackL	*p*-value
Brightness	Back	−15.57	1.69	−2.27	1.60	5.94E-11
	Wing	−13.06	1.64	−8.77	4.49	0.0117
	Comb	−21.65	3.25	−13.74	3.67	0.0001
	Thigh	−6.99	4.99	−3.59	5.87	0.1187
Color-a	Back	1.37	0.45	1.34	1.33	0.9353
	Wing	0.35	0.37	0.34	0.95	0.9728
	Comb	0.18	0.43	1.39	1.63	0.0122
	Thigh	0.39	0.80	0.30	0.57	0.6600
Color-b	Back	0.14	0.85	−0.58	1.61	0.1482
	Wing	−2.41	1.22	−3.08	1.43	0.2739
	Comb	−1.61	1.26	−1.29	1.46	0.5132
	Thigh	3.27	1.67	1.66	1.59	0.0213
Boddweight		2.14	0.19	2.24	0.20	0.1282

### Untargeted metabolome assay

Approximately 0.1 mL of serum was added to a 2.0 mL centrifuge tube containing 400 μL of cold methanol (−20 °C). The mixture was vortexed for 60s and centrifuged at 12,000 g for 10 min at 4 °C. The supernatant from each sample was carefully transferred to a new 2.0 mL centrifuge tube, dried under vacuum, and reconstituted with 150 μL of 80% methanol containing 2-chlorophenylalanine (4 ppm). The samples were filtered through a 0.22 μm membrane for LC-MS analysis. A 20 μL aliquot from each sample was pooled to generate a quality control sample, and the rest was used for LC-MS detection on UltiMate™ 3,000—Q Exactive Plus™ system (ThermoFisher, MA, United States). The detailed conditions are as follows: Chromatographic separation was used with an ACQUITY UPLC^®^ HSS T3 (Waters, MA, United States) column maintained at 40 °C. Gradient elution of analytes was carried out with 0.1% formic acid in water and 0.1% formic acid in acetonitrile for positive ion mode; 5 mM ammonium formate in water and acetonitrile for negative ion mode at a flow rate of 0.25 mL/min. The ESI-MSn run with the spray voltage of 3.5 kV and −2.5 kV in positive and negative ion modes, respectively. Sheath gas and auxiliary gas were set at 30 and 10 arbitrary units. The capillary temperature was 325 °C. Full scans were performed at a resolution of 70,000, with a scan range of m/z 81–1,000, with a collision energy of 30 eV.

### Microbiota DNA extraction and sequencing

The total microbial DNA was extracted from the cecal content using the E.Z.N.A.^®^ Stool DNA Kit (OMEGA, GA, United States) and quantified using the NanoDrop™ 2000 spectrophotometer (ThermoFisher, MA, United States). Sterile water used as negative extraction controls were processed in parallel with biological samples during DNA extraction to monitor potential contamination, and no detectable amplification was observed in these controls. The V3-V4 regions of the bacterial 16S rRNA gene were amplified by PCR with primers 341F (5’-barcode-CCTAYGGGRBGCASCAG) and 806R (5’-barcode-GGACTACNNGGGTATCTAAT). The PCR products were purified by the QIAquick Gel Extraction Kit (Qiagen, MD, United States). Thereafter, amplicon libraries were prepared using the Illumina 16S Metagenomic Sequencing Library Preparation protocol, and sequenced on the NovaSeq6000 (Illumina, CA, United States).

### 16 S rRNA sequence analysis

Raw FASTQ files were processed to exclude adaptor contamination (Cutadapt, V1.9.1) and chimeras (UCHIME Algorithm). High-quality reads were denoised and clustered into amplicon sequence variants (ASVs) using the DADA2 plugin in QIIME2 (v2022.2) against the SILVA_138_SSURef_NR99 reference database. The DADA trimming and truncation parameters were set as follows: –p-trim-left-f 0, –p-trim-left-r 0, –p-trunc-len-f 212, and –p-trunc-len-r 210. Thereafter, alpha diversity and beta diversity were evaluated using QIIME2. Principal coordinates analysis (PCoA) was used to reveal differences in the gut microbiome between groups, and statistical significance was assessed using permutational multivariate analysis of variance (PERMANOVA). Distance-based redundancy analysis (db-RDA) was applied to explain the contribution of skin brightness to gut microbiome grouping. Linear discriminant analysis effect size (LEfSe) and Microbiome multivariable association with linear models 2 (MaAsLin2) were used to identify the different abundance of taxonomy. Functional prediction of microbial communities was performed using Phylogenetic investigation of communities by reconstruction of unobserved states (PICRUSt2) with default settings.

### Metabolome analysis

The obtained raw data were converted into mzXML format (xcms input file format) using Proteowizard software (v3.0.8789). Peak detection, filtration, and alignment were performed using the XCMS package in R (v3.3.2). The main parameters used were bw = 5, ppm = 15, peakwidth = c (5,30), mzwid = 0.015, mzdiff = 0.01, and method = “centWave”. The resulting data matrix contained information on the mass-to-charge ratio (m/z), retention time, and peak intensity for each feature in both positive and negative ionization modes. Data were exported to Excel for further analysis, and batch normalization was performed on the peak intensities to enable comparison of data across different scales. The Orthogonal Partial Least Squares Discriminant Analysis (OPLS-DA) was applied to distinguish the differences in metabolite composition between the two groups, and the random forest (RF) method was used to identify metabolites contributing to group discrimination.

### Statistical analysis

Data analyses were performed using the Wilcoxon rank-sum test for binary variables and to explore differences in α-diversity between two groups. Spearman’s correlation coefficient was applied to assess correlations among samples. Procrustes analysis was performed to assess spatial similarity between gut microbiota and serum metabolite profiles. All figures were generated using R (v3.2.0). Functional prediction and graphical visualization of KEGG pathway information were carried out using STAMP software (v2.1.3). A *p*- < 0.05 was considered statistically significant.

## Results

### Significant differences in skin pigmentation between the BlackD and BlackL groups

Skin color data were collected from 148 Sichuan mountainous black-bone chickens at four anatomical sites: the back, wing, comb, and thigh. From this dataset, the 12 individuals with the darkest and lightest skin brightness were selected and classified into the BlackD and BlackL groups, respectively. Significant differences in skin brightness were observed on the back (*p* = 5.94E-11), wing (*p* = 0.117), and comb (*p* = 0.0001). Apart from a significant difference in color-a for the comb (*p* = 0.0122) and color-b for the thigh (*p* = 0.0213), no other color channels differed significantly between the two groups. Additionally, there was no significant difference in body weight observed between the two groups (Table 1).

### Differences in cecal microbiome composition between chickens with distinct skin brightness

**FIGURE 1:**
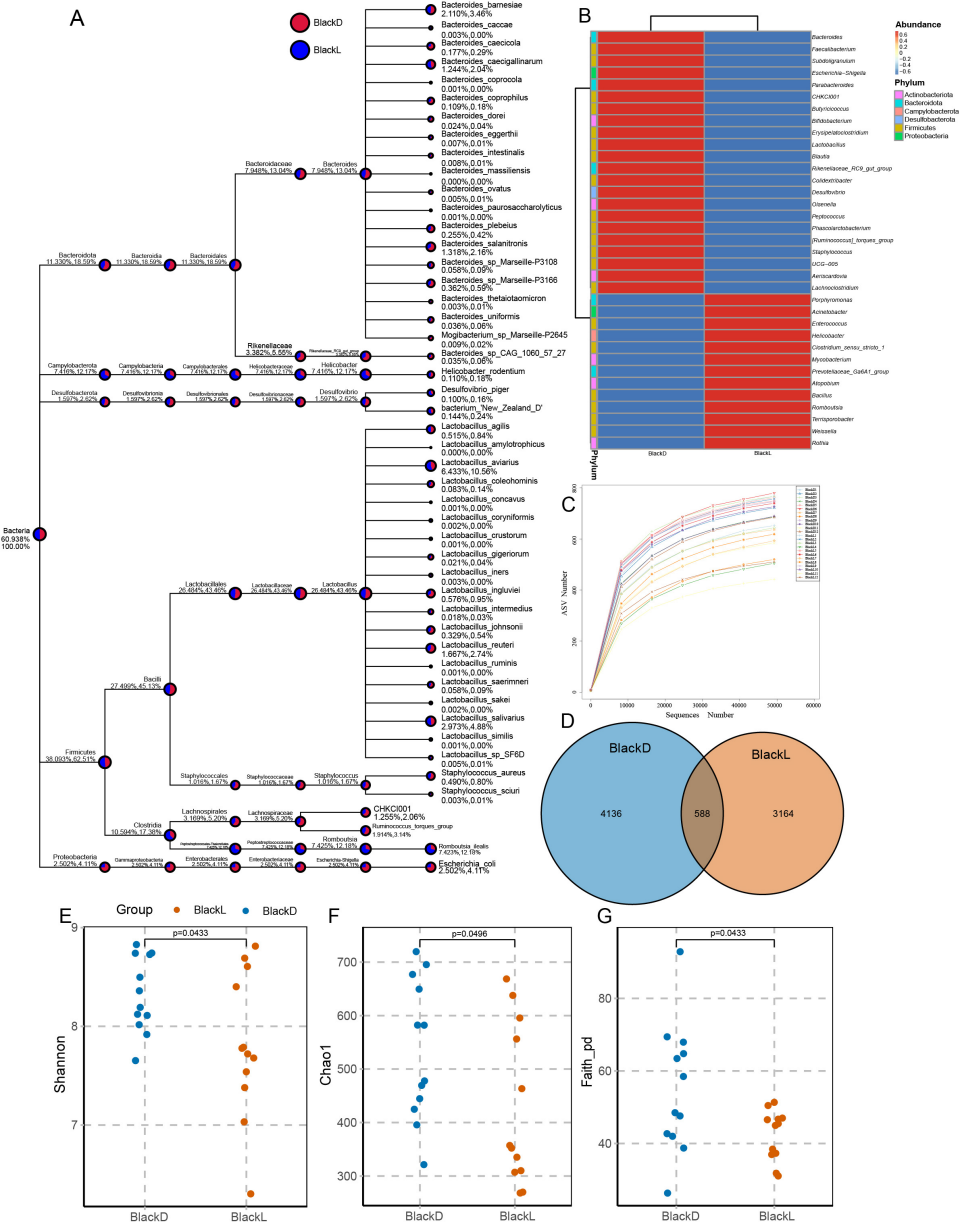
Comparative analysis of gut microbiome abundance and α-diversity in Sichuan mountainous black-bone chickens. Sichuan mountainous black-bone chickens from the BlackD and BlackL groups exhibited distinct microbial characteristics across various taxonomic levels (A). At the genus level, a heatmap revealed significant clustering differences between the two groups (B). In addition, the rarefaction curves gradually plateaued, indicating that sufficient sequencing depth was achieved (C). Out of 6,712 ASVs, 588 were shared between BlackD and BlackL. Additionally, the BlackD group displayed greater α-diversity (D), as evidenced by higher Shannon (E), Chao1 (F), and Faith_pd (G) indices. ASVs, Aamplicon sequence variants; Faith_pd, Faith’s phylogenetic diversity.

An average of 63,488 reads was generated per sample, yielding 6,712 ASVs across all samples. Of these, 588 ASVs were shared between groups, while 4,136 and 3,164 unique ASVs were identified in the BlackD and BlackL groups, respectively. The microbial community in the BlackD group was dominated by the phyla *Firmicutes*, *Bacteroidota*, *Proteobacteria*, *Campylobacterota*, *Actinobacteriota*, *Desulfobacterota*, unidentified_*Bacteria*, *Spirochaetota*, *Fusobacteriota*, and *Synergistota*. The BlackL group exhibited a similar overall phylum composition, but with notable differences in the relative abundance rankings of *Fusobacteriota* and *Spirochaetota*. At the genus level, the top 10 taxa in the BlackD group were *Lactobacillus*, *Bacteroides*, *Helicobacter*, *Rikenellaceae*_*RC9*_*gut*_*group*, *Romboutsia*, *Escherichia*-*Shigella*, [*Ruminococcus*]_*torques*_*group*, *Desulfovibrio*, *Staphylococcus*, and *Faecalibacterium*. In the BlackL group, however, *Bacillus* and *Acinetobacter* replaced *Staphylococcus* and *Faecalibacterium* among the top genera (Figure 1).

**FIGURE 2:**
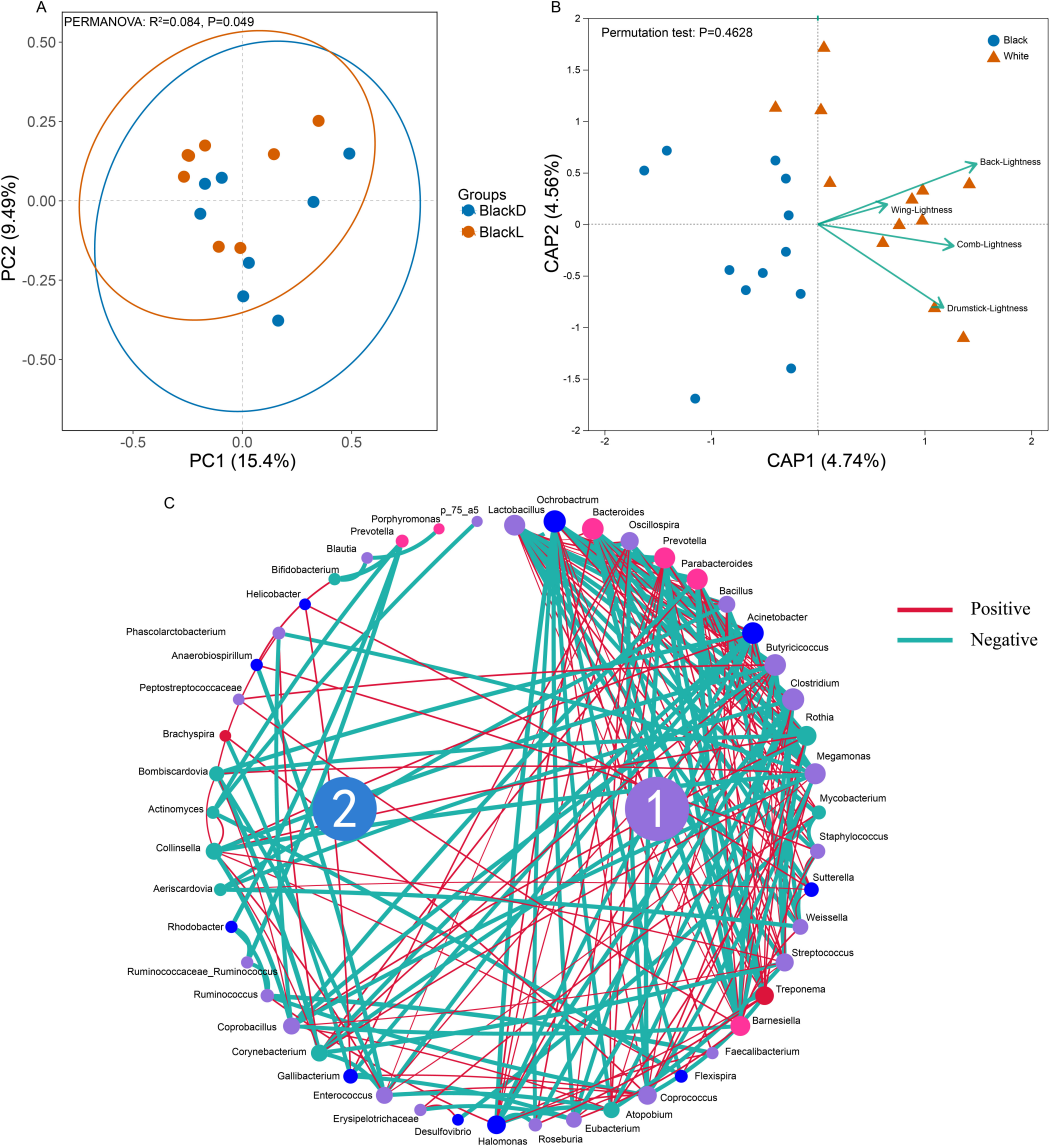
PCoA, db-RDA, and microbial network analysis results in Sichuan mountainous black-bone chickens. Significant differences were observed in the gut microbiota composition between the BlackD and BlackL groups (PERMANOVA, *R*^2^ = 0.084, *P* = 0.049) (A). db-RDA further highlighted the contribution of skin brightness as a variable influencing group separation (B). Network analysis of the gut microbiota revealed intricate interactions, with two distinct zones: a complex Zone 1 and a simpler Zone 2 (C). PCoA: Principal Coordinates Analysis; db-RDA: Distance-based redundancy analysis; PERMANOVA: Permutational multivariate analysis of variance.

Microbial diversity was higher in the BlackD group, as indicated by significantly greater Shannon (*p* = 0.0433), Chao1 (*p* = 0.0496), and Faith’s phylogenetic diversity (Faith_pd, *p* = 0.0496) indices compared with the BlackL group (Figures 1E–G). The significant divergence between the groups was confirmed through PCoA analysis coupled with PERMANOVA, *R*^2^ = 0.084, *P* = 0.049 (Figure 2A). Network analysis revealed intricate interactions within the gut microbiota of Sichuan mountainous black-bone chickens, delineating two distinct regions: Zone 1, characterized by complex relationships, and Zone 2, marked by simpler interactions. For instance, *Peptostreptococcus* exhibited a positive correlation with *Butyricicoccus*, while *Enterococcus* showed positive correlations with both *Butyricicoccus* and *Megamonas* (Figure 2C).

**FIGURE 3:**
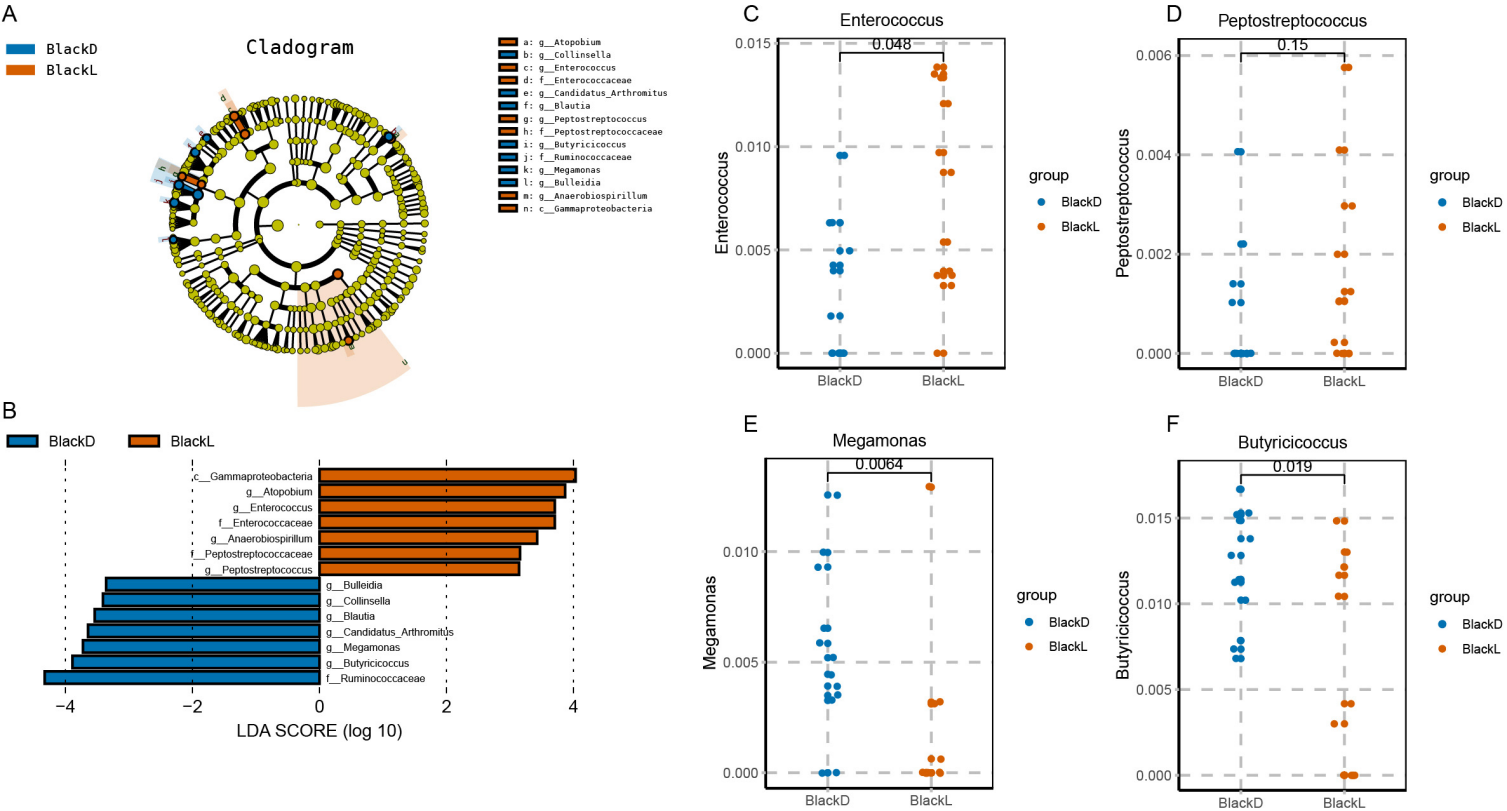
Genus-level differential taxa between BlackD and BlackL groups identified by LEfSe analysis. LEfSe identified significant differences in genera between the BlackD and BlackL groups, including Bulleidia, Collinsella, Blautia, Megamonas, Butyricicoccus, Candidatus Arthromitus, Atopobium, Peptostreptococcus, and Enterococcus (A,B). Notably, genera previously reported to be associated with melanin metabolism were enriched in the BlackL group (C,D). In contrast, genera linked to energy metabolism were enriched in the BlackD group (E,F). LEfSe: Linear discriminant analysis effect size.

LEfSe analysis identified distinct taxonomic signatures enriched in each group. At the genus level, *Bulleidia*, *Collinsella*, *Blautia*, *Megamonas*, *Butyricicoccus*, and *Candidatus_Arthromitus* were significantly more abundant in the BlackD group (Kruskal-Wallissum-rank test, LDA ≥ 3, *p* < 0.05). Conversely, *Atopobium*, *Enterococcus*, *Anaerobiospirillum*, and *Peptostreptococcus* were enriched in the BlackL group (Kruskal-Wallissum-rank test, LDA ≥ 3, *p* < 0.05). Surprisingly, genera previously reported to be associated with L-tyrosine or melanin metabolism, such as *Enterococcus* (LDA = 3.707, *p* = 0.0154) and *Peptostreptococcus* (LDA = 3.14, *p* = 0.0461), were more abundant in the BlackL group rather than the BlackD group (Figure 3). Additionally, genera associated with energy metabolism, such as *Megamonas* (LDA = 3.72, *p* = 0.0157) and *Butyricicoccus* (LDA = 3.89, *p* = 0.0150), were markedly enriched in the BlackD group.

### Enrichment of energy metabolism-related pathways in the BlackD group based on functional metagenomic prediction

PICRUSt2 analysis identified 35 out of 345 taxonomy-associated KEGG pathways and 25 of 415 MetaCyc pathways as differentially represented between groups. Interestingly, no tyrosine metabolism-related pathways were enriched in the BlackD group according to PICRUSt2 predictions. Instead, multiple energy metabolism-related pathways were significantly enriched in the BlackD group.

Twenty-five predicited MetaCyc pathways were associated with amino acid and nitrogen metabolism, carbohydrate metabolism, lipid and cell wall metabolism, nucleotide metabolism, and other metabolic processes, all showing significant differences between the BlackD and BlackL groups. This indicated that the microbiome in the BlackD group exhibited a higher level of energy metabolism activity (Table 2).

**TABLE 2:** Differential metabolic MetaCyc pathways of gut microbiome predicted by PICRUSt2.

Pathway ID	Pathway name	Mean-BlackD	SE-BlackD	Mean-BlackL	SE-BlackL	*P*-value	Bonferroni adjusted p	Type of metabolism
Daplysinesyn-PWY	L-lysine biosynthesis I	0.0051	0.0003	0.0054	0.0005	0.0141	0.0141	Amino acid and nitrogen metabolism
PWY-5505	L-glutamate and L-glutamine biosynthesis	0.0024	0.0005	0.0019	0.0005	0.0141	0.0141	
PWY-6478	GDP-D-glycero-α-D-manno-heptose biosynthesis	0.0004	0.0001	0.0002	0.0001	0.0217	0.0217	
PWY-6629	Superpathway of L-tryptophan biosynthesis	0.0011	0.0008	0.0007	0.0005	0.0416	0.0416	
GALACT-GLUCUROCAT-PWY	Superpathway of hexuronide and hexuronate degradation	0.0016	0.0003	0.0013	0.0003	0.0367	0.0367	Carbohydrate and energy metabolism
GALACTUROCAT-PWY	D-galacturonate degradation I	0.0025	0.0005	0.0021	0.0006	0.0416	0.0416	
GLUCARDEG-PWY	D-glucarate degradation I	0.0005	0.0002	0.0003	0.0001	0.0188	0.0188	
GLUCUROCAT-PWY	Superpathway of beta-D-glucuronosides degradation	0.0016	0.0003	0.0014	0.0003	0.0248	0.0248	
P341-PWY	Glycolysis V (Pyrococcus)	3.44E-05	1.00E-04	1.61E-05	1.00E-04	0.0261	0.0261	
P163-PWY	L-lysine fermentation to acetate and butanoate	0.0007	0.0003	0.0005	0.0003	0.0065	0.0065	
PWY-7332	Superpathway of UDP-N-acetylglucosamine-derived O-antigen building blocks biosynthesis	0.0008	0.0005	0.0005	0.0004	0.0248	0.0248	
GLYCOCAT-PWY	Glycogen degradation I	0.0058	0.001	0.0049	0.0013	0.0217	0.0217	
PWY-6737	Starch degradation V	0.0057	0.0012	0.0048	0.0014	0.0188	0.0188	Lipid and cell wall metabolism
LPSSYN-PWY	Superpathway of Kdo2-lipid A biosynthesis	0.0005	0.0003	0.0003	0.0002	0.0188	0.0188	
PWY-5188	Tetrapyrrole biosynthesis I (from glutamate)	0.0034	0.0005	0.0039	0.0009	0.0367	0.0367	
PWY-5189	Tetrapyrrole biosynthesis II (from glycine)	0.0031	0.0005	0.0036	0.0009	0.0416	0.0416	Nucleotide Metabolism
1CMET2-PWY	Folate transformations III (*E. coli*)/N10-formyl-tetrahydrofolate biosynthesis	0.0055	0.0006	0.0051	0.0007	0.0284	0.0284	
PWY-5507	Adenosylcobalamin biosynthesis I (anaerobic)	0.0011	0.0004	0.0015	0.0004	0.0217	0.0217	
PWY-7377	Cob(II)yrinate a,c-diamide biosynthesis I (early cobalt insertion)	0.0013	0.0005	0.0017	0.0006	0.0416	0.0416	
METH-ACETATE-PWY	Methanogenesis from acetate	0.0011	0.0007	0.0019	0.0011	0.0416	0.0416	Other metabolism
PWY-5676	Acetyl-CoA fermentation to butanoate	0.0021	0.0003	0.0014	0.0004	0.0002	0.0002	
PWY-5677	Succinate fermentation to butanoate	0.0007	0.0002	0.0004	0.0002	0.0016	0.0016	
PWY-6588	Pyruvate fermentation to acetone	0.0025	0.0003	0.0018	0.0004	0.0002	0.0002	
PWY-6749	CMP-legionaminate biosynthesis I	0.0005	0.0002	0.0003	0.0002	0.0217	0.0217	
PWY-7392	Taxadiene biosynthesis (engineered)	0.0043	0.0003	0.0041	0.0004	0.047	0.0470	

**FIGURE 4:**
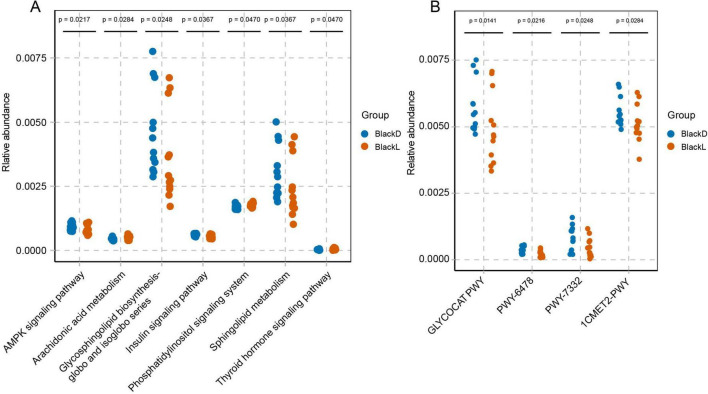
KEGG and MetaCyc pathway predictions based on PICRUSt2. Functional predictions at KEGG Level 3 (A) and MetaCyc (B) levels indicated that pathways closely associated with energy metabolism were significantly enriched in the BlackD group. PICRUSt2: Phylogenetic investigation of communities by reconstruction of unobserved states.

Additionally, seven predicted KEGG pathways were significantly enriched in the gut microbiome of the BlackD group, including the AMPK signaling pathway, phosphatidylinositol signaling system, glycosphingolipid biosynthesis (globo and isoglobo series), arachidonic acid metabolism, sphingolipid metabolism, insulin signaling pathway, and thyroid hormone signaling pathway. These pathways, particularly the AMPK signaling pathway, insulin signaling pathway, and thyroid hormone signaling pathway, are closely linked to energy metabolism and showed higher abundance in the BlackD group (Figure 4).

These findings further support the conclusion that the intestinal microbiome of the BlackD group is characterized by enhanced energy metabolism, which may indirectly facilitate melanin synthesis through increased metabolic energy supply.

### Serum metabolites associated with the skin brightness in Sichuan mountainous black-bone chickens

**FIGURE 5:**
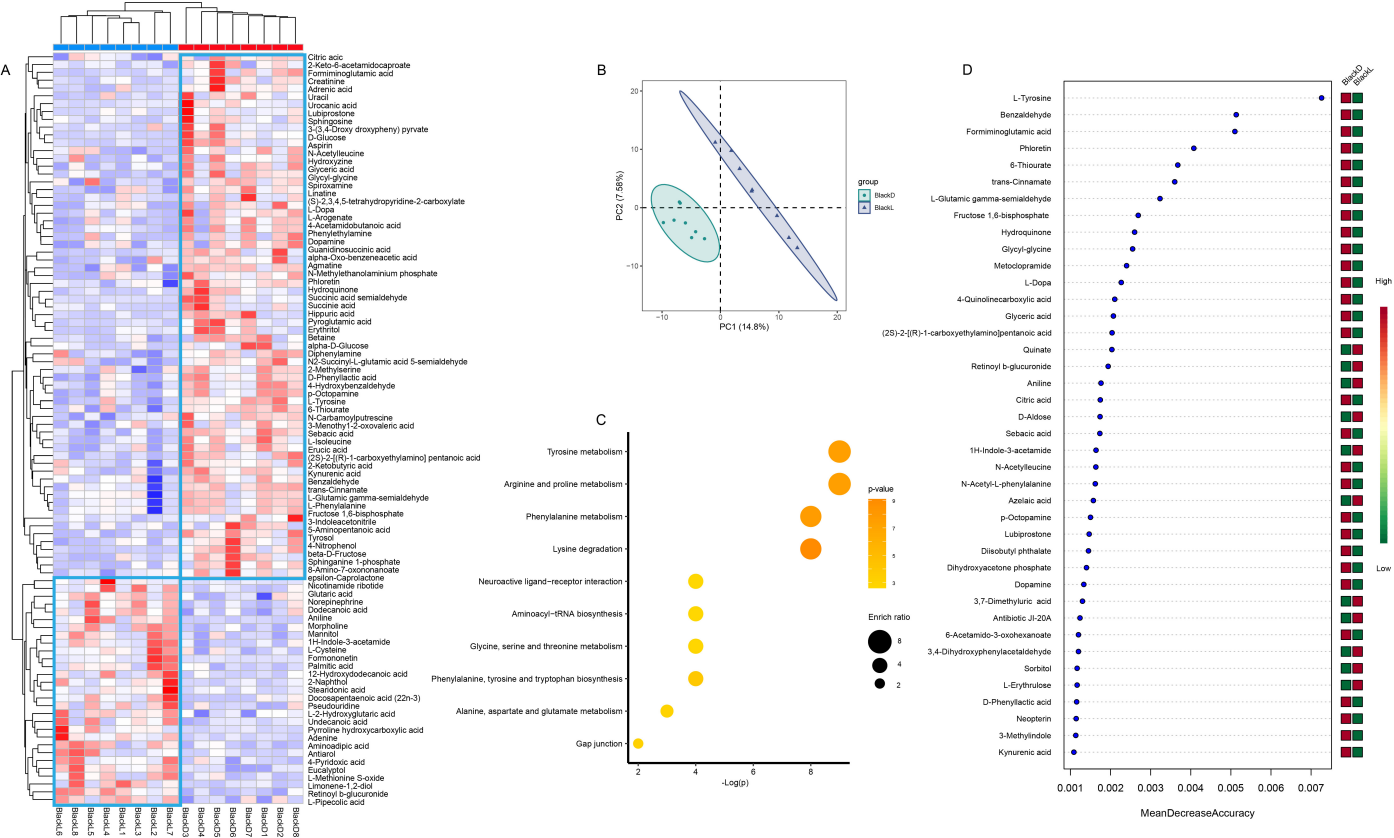
Differential analysis of serum metabolome. Spearman’s correlation analysis of the serum metabolome revealed distinct metabolic profiles between the two groups (A), with OPLS-DA further confirming significant differences (B). The top 10 enriched KEGG pathways were closely related to amino acid metabolism, including the tyrosine metabolism pathway (C). Random forest analysis identified differential metabolites, with those associated with melanin and energy metabolism being significantly enriched in the BlackD group (D). OPLS-DA: orthogonal partial least squares discriminant analysis.

Untargeted metabolomics identified 1,786 serum metabolites across all samples. These metabolites exhibited different metabolic profiles between the two groups, with metabolites related to the melanin metabolism pathway showing a significantly higher abundance in the BlackD group. Notably, metabolites related to tyrosine and melanin metabolism, such as L-Tyrosine, L-DOPA, dopamine, L-phenylalanine, 4-hydroxybenzaldehyde, and hydroquinone, were significantly elevated in BlackD (*p* < 0.05) (Figure 5A). The OPLS-DA further confirmed significant differences between the two groups (*p* = 0.001) (Figure 5B).

Subsequently, we screened 250 metabolites from the OPLS-DA analysis with variable importance in projection score > 1.5 and *p* < 0.05. The RF model was applied to assess their contributions to group classification. Metabolites closely linked to tyrosine or melanin metabolism demonstrated greater contributions in the BlackD group, with L-tyrosine exhibiting the highest contribution (Figures 5C, D).

**FIGURE 6:**
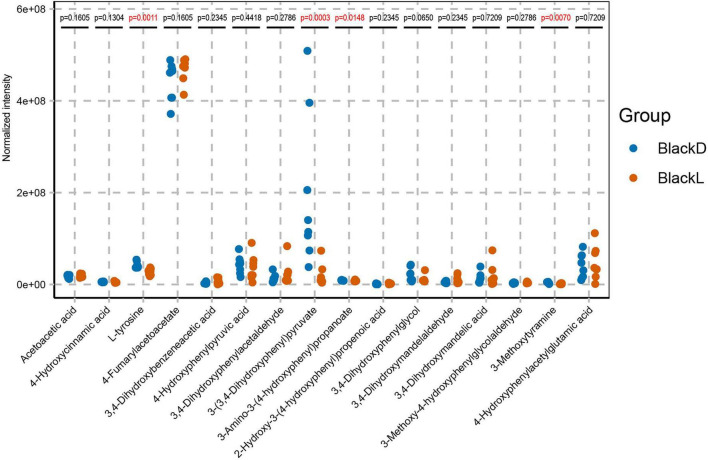
Comparison of metabolites abundance in tyrosine metabolism-related pathways.

Furthermore, these differentially expressed metabolites were mapped to 61 KEGG pathways, among which six showed significant differences (*p* < 0.05): lysine degradation, phenylalanine metabolism, arginine and proline metabolism, tyrosine metabolism, phenylalanine, tyrosine, and tryptophan biosynthesis, and gap junction. We focused particular attention on the tyrosine metabolism pathway, given its central role in melanogenesis. This pathway encompassed 16 distinct compounds: acetoacetic acid, 4-hydroxycinnamic acid, L-tyrosine, 4-fumarylacetoacetate, 3,4-dihydroxybenzeneacetic acid, 4-hydroxyphenylpyruvic acid, 3, 4-dihydroxyphenylacetaldehyde, 3-(3,4-dihydroxyphenyl) pyru- vate, 3-amino-3-(4-hydroxyphenyl) propanoate, 2-hydroxy-3-(4-hydroxyphenyl) propenoic acid, 3,4-dihydroxyphenylglycol, 3,4-dihydroxymandelaldehyde, 3,4-dihydroxymandelic acid, 3- methoxy-4-hydroxyphenylglycolaldehyde, 3-methoxytyramine, and 4-hydroxyphenylacetylglutamic acid. Further abundance analysis revealed that L-tyrosine (*p* = 0.0011), 3-(3,4-dihydroxyphenyl) pyruvate (*p* = 0.0003), 3-amino-3-(4-hydroxyphenyl) propanoate (*p* = 0.0148), and 3-methoxy-4-hydroxyphenylglycolaldehyde (*p* = 0.0070) were significantly more abundant in the BlackD group (Figure 6).

Additionally, among the metabolites associated with energy metabolism identified through RF analysis, including Fructose 1,6-bisphosphate, Dihydroxyacetone phosphate, Citric acid, and Glyceric acid, all exhibited significantly higher abundance in the BlackD group (Figure 5D). These results collectively support the hypothesis that the enhanced energy metabolism of the BlackD microbiome may facilitate melanin biosynthesis through energy-dependent processes.

### Correlation between gut microbiota and serum metabolites in Sichuan mountainous black-bone chickens

To elucidate the potential relationship between gut microbiome and serum metabolites in Sichuan mountainous black-bone chickens, we conducted a correlation analysis between differential metabolites, differential microbial taxa, and skin brightness in Sichuan mountainous black-bone chickens. The db-RDA analysis confirmed that skin brightness (back, wing, comb, and thigh) was the primary explanatory factor driving microbial variation (Figure 2B).

**FIGURE 7:**
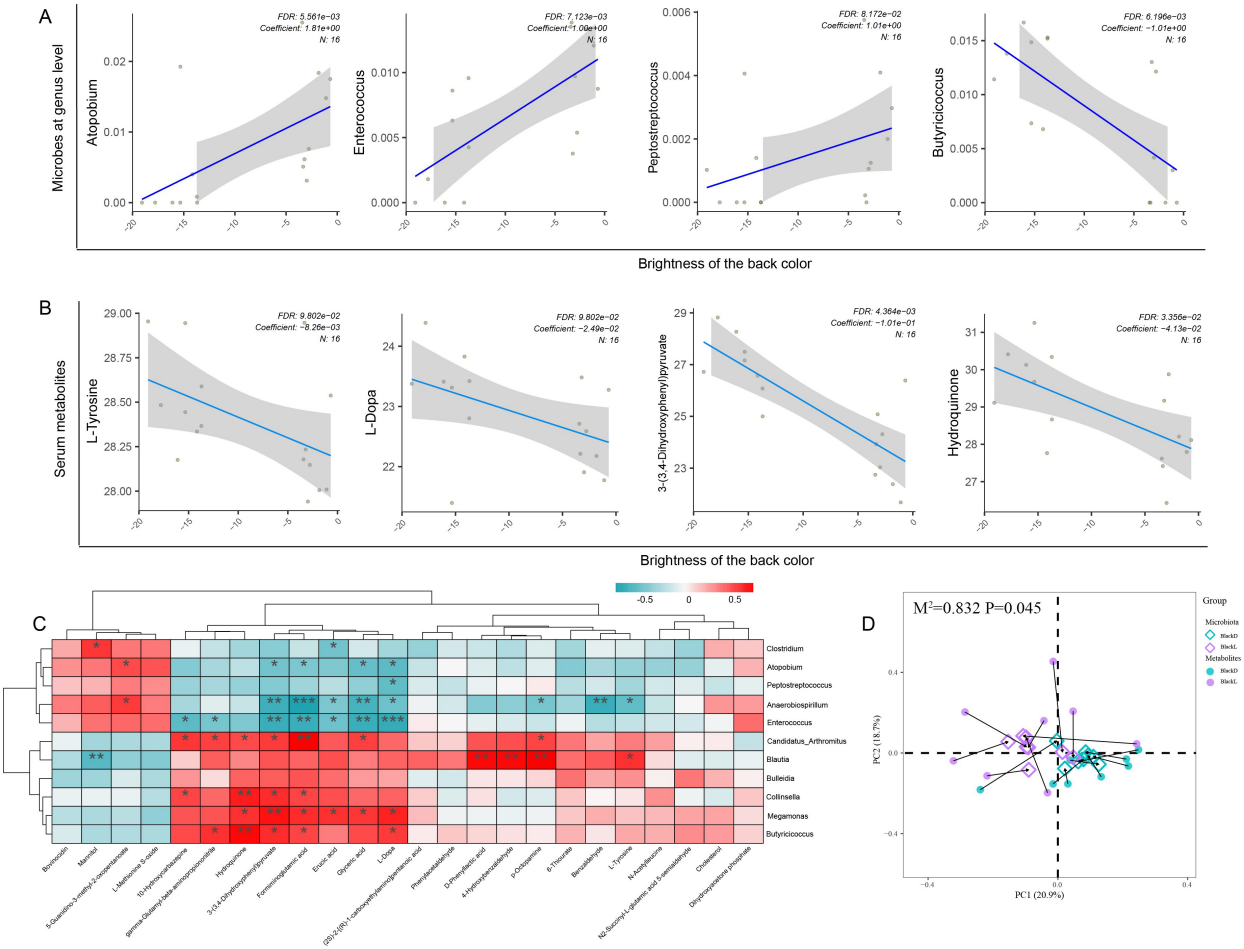
Correlation analysis of differential genera and metabolites. Spearman’s correlation analysis demonstrated relationships between differential metabolites and genera (A). Serum metabolites related to melanin metabolism exhibited a significant negative correlation with the brightness of skin on the back (B). In the gut microbiota, genera previously reported to be associated with melanin metabolism showed a positive correlation with the brightness of skin on the back, while genera closely linked to energy metabolism displayed a negative correlation with skin brightness (C). The Procrustes analysis demonstrated the spatial similarity between gut microbiota and serum metabolites with a *M*^2^ = 0.832 and *p* = 0.045 (D). *P < 0.05, **P < 0.01, ***P < 0.001.

MaAslin2 analysis revealed that genera previously associated with tyrosine or melanin metabolism, such as *Atopobium*, *Enterococcus*, *Candidatus*_*Arthromitus*, *Blautia*, and *Clostridium*, were positively correlated with the skin brightness of Sichuan mountainous black-bone chickens. In contrast, genera linked to short-chain fatty acid (SCFA) metabolism, including Butyricicoccus and Megamonas, exhibited a negative correlation with skin brightness (Figure 7A). The result of Procrustes analysis based on PCA showed a significant correlation between the microbial community and serum metabolite profiles (*M*^2^ = 0.832, *P* = 0.045). This correlation remained consistent when analysis was extended to species-level taxonomic resolution (Supplementary Figure 1).

We also observed that numerous metabolites associated with amino acid metabolism, carbohydrate metabolism, and lipid metabolism were negatively correlated with the skin brightness. Specifically, metabolites closely tied to tyrosine and melanin metabolism, such as L-Tyrosine, L-Dopa, 3-(3,4-Dihydroxyphenyl) pyruvate, 4-Hydroxybenzaldehyde, and Hydroquinone, showed a negative correlation with brightness. In other words, darker skin coloration in black-bone chickens corresponded to higher abundances of these metabolites (Figure 7B).

Correlation analysis between differential microbial genera and metabolites further revealed that Butyricicoccus and Megamonas (associated with SCFA and energy metabolism) were positively correlated with melanin-related metabolites such as L-tyrosine, L-DOPA, 3-(3,4-dihydroxyphenyl) pyruvate, 4-hydroxybenzaldehyde, and hydroquinone. Conversely, *Enterococcus* and *Peptostreptococcus* displayed a negative correlation with these tyrosine- and melanin-related metabolites (Figure 7C).

## Discussion

This study represents the first comprehensive characterization of the gut microbiome and serum metabolome in Sichuan mountainous black-bone chickens with distinct skin pigmentation, and the first to explore their potential interrelationship with melanin deposition. Significant compositional and functional differences were observed between the BlackD and BlackL groups. Only 588 of 6,712 ASVs were shared between groups, and the BlackD group exhibited higher microbial diversity. Untargeted metabolomic analysis revealed that BlackD chickens possessed distinct serum metabolic profiles, characterized by higher abundances of melanin-related metabolites. Notably, gut microbiome and serum metabolite patterns in dark-skinned chickens demonstrated correlations with enhanced energy metabolism, suggesting that gut microbiome may facilitate melanin deposition by modulating host metabolic activity.

Recent evidence has highlighted the role of the gut microbiome in shaping host phenotypes. For instance, color variants of Oujiang color common carp with TYRP1 and SCARB1 mutations display distinct microbial community structures, with altered abundances of *Acinetobacter*, *Chryseobacterium*, *Macrococcus*, *Bacillus*, and *Pseudomonas* (Kanika et al., 2024; Nathan Mandal et al., 2024). Similarly, studies on the gut–skin axis have revealed strong associations between microbial composition and skin conditions in humans, such as atopic dermatitis, where *Akkermansia*, *Bacteroides*, and *Bifidobacterium* are reduced, while *Faecalibacterium*, *Clostridium*, and *Escherichia* are elevated (De Pessemier et al., 2021; Lee et al., 2016; Song et al., 2016). Bacteria such as *Bacillus*, *Amorphothica*, *Pseudomonas*, *Burkholderia*, and *Stenotrophomonas* have been reported to participate in melanin metabolism (Singh et al., 2021). In our study, we did not observe an enrichment of previously reported melanin-related bacterial genera in the BlackD group but instead in the BlackL group. This finding suggests that darker-skinned chickens may already possess sufficient melanin intermediates, reducing the need for microbial production of melanin precursors. Rather, increased energy may be required to facilitate biosynthesis and the intercellular transport of melanin across multicellular compartments (Snyman et al., 2024). Consistent with this interpretation, network analysis revealed positive correlations between *Megamonas* and *Butyricicoccus* linked to SCFA metabolism and energy-related bacterial genera. Furthermore, the elevated levels of melanin-related metabolites observed in the serum metabolome of the BlackD group support this hypothesis. The precise mechanisms underlying these observations warrant further investigation.

We found that the BlackD group was enriched in bacterial genera associated with energy metabolism, including *Bulleidia*, *Collinsella*, *Blautia*, *Megamonas*, *Butyricicoccus*, and *Candidatus*_*Arthromitus* (Eeckhaut et al., 2013; Qin et al., 2019; Xiao et al., 2025; Zhang et al., 2025). In contrast, no genera directly involved in melanin biosynthesis were differentially abundant. Pathway analysis further revealed higher levels of energy metabolism pathways in BlackD chickens, with *Megamonas* and *Butyricicoccus* closely linked to SCFA metabolism (Xiao et al., 2025; Zhang et al., 2025). This pattern is consistent with findings from comparative studies of Silky Fowl and White Leghorn chickens, where genes related to lipid metabolism and melanogenesis were significantly upregulated in the Silky Fowl cecum, while genes associated with glucose and bile acid metabolism were more highly expressed in White Leghorns (Yang et al., 2022). Moreover, predicted KEGG pathways, such as the AMPK signaling pathway (Bang et al., 2017), insulin signaling pathway (D’Mello et al., 2016), and thyroid hormone signaling pathway (Zhao et al., 2023), showed higher predicted functional potential in the BlackD group. These pathways regulate oxidative stress and energy metabolism to influence melanogenesis. Specifically, the AMPK signaling pathway plays a crucial role in adiponectin-mediated melanin formation in melanocytes (Bang et al., 2017). Under hypoglycemic or hypoxic conditions, AMPK, along with insulin and thyroid hormone signaling pathways, enhances glucose uptake and utilization while reducing fatty acid and protein synthesis, ultimately supporting melanin biosynthesis (Edmunds et al., 2019; Li et al., 2019). Our results align with these mechanisms, indicating that the gut microbiome in dark-skinned chickens may enhance melanin deposition through energy metabolic activity.

Tyrosine metabolism is the biochemical core of melanin synthesis (Snyman et al., 2024). In this study, metabolites related to this pathway, such as L-tyrosine, L-DOPA, and dopaquinone, were significantly higher in the serum of BlackD chickens, consistent with increased melanin deposition. Interestingly, Spearman’s correlation analysis showed that gut bacteria associated with energy metabolism, including *Megamonas* and *Butyricicoccus*, positively correlated with melanin-related metabolites like L-tyrosine, L-DOPA, and dopaquinone. Studies have shown that the gut microbiome can directly influence host serum metabolomics by modulating postprandial triglyceride and insulin levels (Asnicar et al., 2021). For example, *Prevotella copri* is associated with improved cardiometabolic profiles and reduced visceral fat accumulation. Similarly, L-tyrosine levels have been reported to correlate positively with *Pseudomonas*, *Kaistia*, *Cetobacterium*, *Enterococcus*, *Galbitalea*, *Legionella*, *Neochlamydia*, and Timonella, while showing negative correlations with Bacteroides, Achromobacter, and *Acinetobacter* (Asnicar et al., 2021). Sultan et al. (Sultan et al., 2022) demonstrated that melanin deposition relies heavily on mitochondrial fatty acid oxidation. SREBF1 is highly active during melanogenesis, ensuring energy supply through fatty acid β-oxidation (Li et al., 2019). Consistent with this, our functional predictions identified enrichment of the fatty acid β-oxidation pathway (PWY-7332) in the BlackD group (Gong et al., 2022), alongside other energy-related pathways (P163-PWY, P341-PWY, and GALACT-GLUCUROCAT-PWY) involved in carbohydrate and lipid metabolism (Deng et al., 2021; Han et al., 2024; Liu et al., 2025). Additionally, research shows that (-)-Hydroxycitric acid activates the adiponectin-AMPK signaling pathway, enhancing glycolysis, reducing fat droplet accumulation in chickens, and accelerating energy metabolism (Li et al., 2019). These findings align with our results, as energy metabolism-related pathways (AMPK signaling, insulin signaling, and thyroid hormone signaling pathways) were significantly enriched in BlackD chickens compared to BlackL chickens, reinforcing the idea that the gut microbiome may associate with melanin deposition through metabolic activity.

Although this study revealed significant associations among the gut microbiome, energy metabolism, and melanin deposition, causal relationships remain to be experimentally validated. Future studies employing fecal microbiota transplantation or metabolite intervention experiments are needed to confirm the functional role of these microbial communities in melanin biosynthesis. More importantly, isotopic tracer techniques or germ-free animal models could be applied in future research to distinguish microbial-derived metabolites from those produced by the host itself. Such approaches would help to clarify the direct metabolic contributions of gut microbes, thereby providing a more precise understanding of the microbiome–host metabolic interplay underlying melanin deposition. In addition, several limitations should be noted. Although significant differences in gut microbial composition were detected between dark- and light-skinned chickens, the PERMANOVA result (*R*^2^ = 0.084, *P* = 0.049) indicates a modest effect size, suggesting subtle rather than pronounced microbiome shifts. On the other hand, a smaller sample size could also be another reason for the weak effect of PERMANOVA. Meanwhile, the limited sample size and single breeding source may constrain the generalizability of our findings. Finally, as this study is correlative and the function is based on PIRCUST2 prediction, further experiments involving microbiota manipulation or gene expression validation are needed to clarify causal links between microbial metabolism and melanin deposition.

## Conclusion

This study provides novel insights into the interaction between the gut microbiome and host pigmentation in Sichuan mountainous black-bone chickens. Rather than directly participating in melanin biosynthesis, the gut microbiome appears to influence melanin deposition indirectly by modulating host energy metabolism. These findings highlight a potential microbiome–energy–melanin axis that may underlie pigmentation diversity in poultry, offering a promising avenue for targeted breeding and nutritional interventions to enhance the economic value of black-bone chickens.

## Data Availability

The data presented in this study are publicly available. The data can be found at: https://www.ncbi.nlm.nih.gov/, accession PRJNA1237538.

## Supplementary material

Supplementary Figure S1 Correlation analysis of species-level microbial abundance with skin lightness. Spearman’s correlation analysis demonstrated relationships between differential skin lightness and species. *Enterococcus_cecorum*, *Clostridium_ruminantium*, *Anaerobiospirillum_thomasii*, *Eubacterium__cylindroides*, and *Peptostreptococcus_anaerobius* were positively associated with skin lightness. *Megamonas_hypermegale*, *Butyricicoccus_pullicaecorum*, and *Faecalibacterium_prausnitzii* were negatively associated with skin lightness.
