# Supplementary figures and images for: Gut microbiome associated with melanin deposition by supporting energy metabolism in Sichuan mountainous black-bone chickens
Source: Front Microbiol. 2025 Dec 15;16:1682376. doi: 10.3389/fmicb.2025.1682376 (PMC12745435; doi:10.3389/fmicb.2025.1682376)

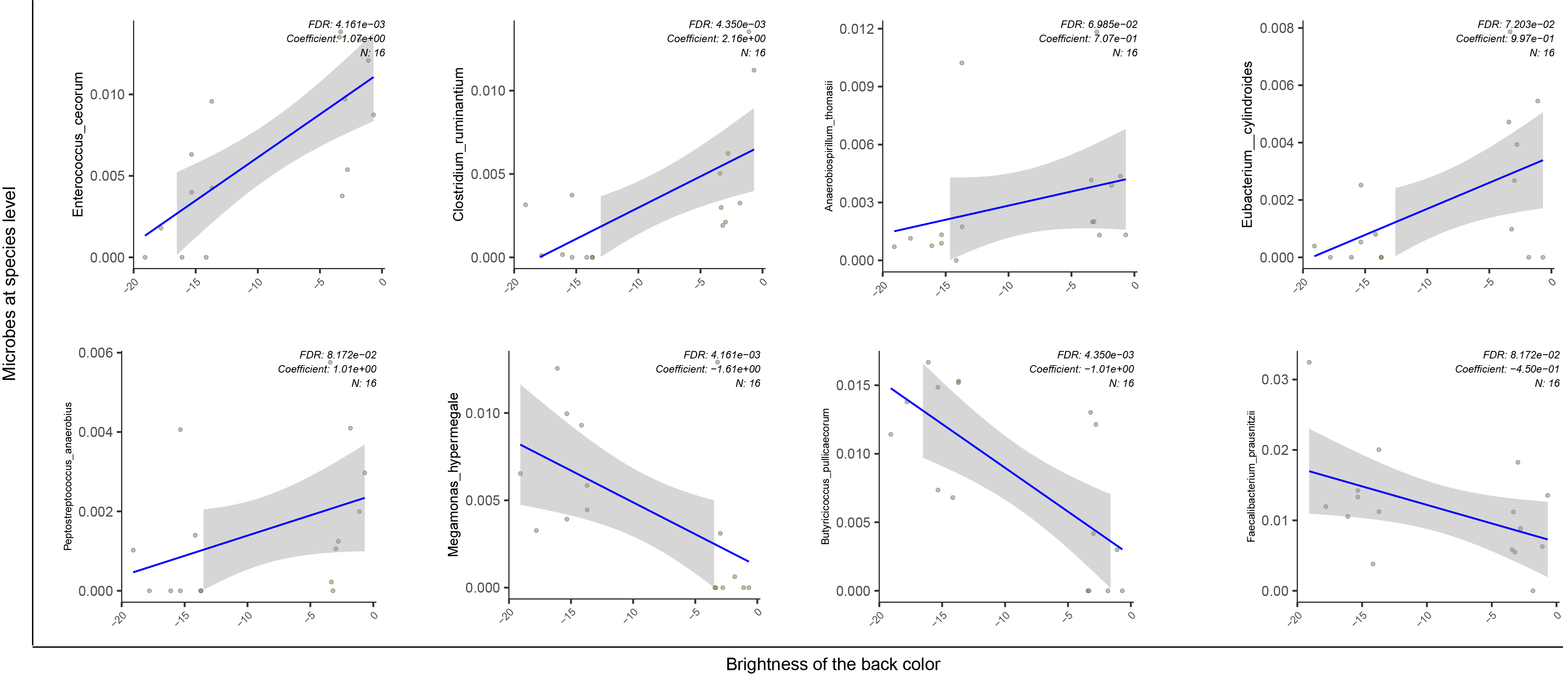

Supplement: Supplementary Figure S1 — Correlation analysis of species-level microbial abundance with skin lightness. Spearman’s correlation analysis demonstrated relationships between differential skin lightness and species. Enterococcus_cecorum, Clostridium_ruminantium, Anaerobiospirillum_thomasii, Eubacterium__cylindroides, and Peptostreptococcus_anaerobius were positively associated with skin lightness. Megamonas_hypermegale, Butyricicoccus_pullicaecorum, and Faecalibacterium_prausnitzii were negatively associated with skin lightness. [file Image_1.jpeg]
